# Carbapenem-resistant *Salmonella* Derby harboring a plasmid carrying *bla*_NDM-1_ from a clinical case in China

**DOI:** 10.3389/fcimb.2026.1765519

**Published:** 2026-03-09

**Authors:** Mengyuan Wang, Chunhua Han, Mingju Hao, Wenxue Zhang, Shifu Wang

**Affiliations:** 1 Department of Microbiology Laboratory, Children's Hospital Affiliated to Shandong University, Jinan, China; 2 Department of Microbiology Laboratory, Jinan Children's Hospital, Jinan, China; 3 Department of Clinical Laboratory, The Affiliated Hospital of Qingdao University, Qingdao, China; 4 Department of Clinical Laboratory, Shandong Provincial Qianfoshan Hospital, Jinan, China

**Keywords:** *bla*_NDM-1_, carbapenem resistance, conjugative plasmid, non-typhoidal *Salmonella*, *S.* Derby

## Abstract

**Objective:**

The increasing antimicrobial resistance in non-typhoidal *Salmonella* (NTS) poses a growing challenge to clinical therapy. This study reports, for the first time, a carbapenem-resistant *Salmonella enterica* serovar Derby isolate. Although serovar Derby accounts for a relatively small proportion of clinical NTS infections, elucidating the mechanism, origin, and dissemination potential of its carbapenem resistance is crucial for enhancing surveillance and prevention strategies against resistant NTS.

**Methods:**

Antimicrobial susceptibility testing was performed using commercial broth microdilution panels with the Beckman Coulter WalkAway 96 PLUS system. Whole-genome sequencing (WGS) and S1-pulsed-field gel electrophoresis (PFGE) were employed to characterize the chromosomes and plasmids of isolates. Conjugation assays were conducted to evaluate plasmid mobility. Additionally, the NCBI Genome and Pathogens databases were used to identify carbapenemase-producing *Salmonella* strains.

**Results:**

A patient with aplastic anemia was admitted with abdominal pain and received successive treatments. During periods of recurrent fever, carbapenem-resistant *S.* Derby (CS_CRSA) and *Escherichia coli* (CS_CREco) were isolated from rectal swabs. WGS revealed that both strains carried a nearly identical IncFII plasmid (80,195/80,198 bp) harboring *bla*_NDM-1_ and *qnrS1* genes. This plasmid contained a complete conjugation module, and could be transferred from CS_CRSA and CS_CREco to the recipient at efficiencies of (4.50 ± 1.29)×10^−2^ and (3.17 ± 0.74)×10^−1^. Comparative analysis showed its high similarity to a resistance plasmid of *Salmonella enterica* serovar Typhimurium isolated from Zhejiang, China. As of June 25, 2025, 35 fully assembled *Salmonella enterica* strains carrying carbapenemase genes were identified, predominantly *S.* Typhimurium and its variants. Phylogenetic analysis indicated that most carbapenemase-producing *Salmonella* (CPSA) strains were scattered, while clonal dissemination was observed in some serotypes.

**Conclusion:**

This study reports a clinical isolate of carbapenem-resistant *S.* Derby, likely resulting from horizontal transfer of a *bla*_NDM-1_*-*carrying plasmid, which indicates that carbapenem resistance is extending to less common and low virulence serovars of *Salmonella*. The emergence of such strains poses a challenge to patient care, especially for immunocompromised populations suffering from invasive infections. Additionally, clonal dissemination of CPSA in certain serotypes warrants heightened vigilance and preventive measures.

## Introduction

*Salmonella* is a highly ubiquitous foodborne pathogen that causes infections or colonization in various hosts. Typhoidal *Salmonella* (TS) serovars often cause lethal systemic infections (GBD 2017 Typhoid and Paratyphoid Collaborators, 2019; Syed et al., 2020), while the disease caused by non-typhoidal *Salmonella* (NTS) should not be overlooked. In recent years, the incidence of NTS infections and the frequency of extraintestinal complications have continued to rise (Marchello et al., 2022; Ke et al., 2024a). The scale of its prevalence and disease burden has gradually surpassed that of TS, establishing NTS as the predominant type of *Salmonella* infection (Marchello et al., 2022; Hancuh et al., 2023). In 2019, the global death toll from NTS infections reached 215,000, with 87,100 fatalities attributed to bloodstream infections and 79,100 to other invasive infections, surpassing the 182,000 deaths caused by TS (GBD 2019 Antimicrobial Resistance Collaborators, 2022). The extensive use and exposure to antimicrobials have exacerbated the emergence and spread of antimicrobial-resistant NTS (Zhao et al., 2024). The resistance to frontline agents like fluoroquinolone and third-generation cephalosporins is now widespread and severe (Rosso et al., 2023; Zhou et al., 2023; Wang et al., 2025), leading the World Health Organization (WHO) to designate fluoroquinolone-resistant NTS as a high-priority pathogen (Sati et al., 2025). Against this backdrop, carbapenems have become the last-resort therapy for severe, multidrug-resistant infections of NTS. However, the recent rise of carbapenem-resistant *Salmonella* (CRSA) poses a new and significant challenge for the clinical management of *Salmonella* infections (Deng et al., 2024; Song et al., 2025).

Current surveillance and reports on CRSA have primarily focused on high-incidence serovars, such as Typhimurium (Feng et al., 2025), Enteritidis (Cordeiro et al., 2025), Goldcoast (Wu et al., 2023), and London (Tan et al., 2024). However, a certain knowledge gap exists regarding whether carbapenem resistance has spread to other clinically less common but epidemiologically important serovars. Understanding whether and how carbapenem resistance emerges in these serovars is crucial for informing comprehensive surveillance strategies. *Salmonella enterica* serovar Derby is a prime example of such a serovar. Although its clinical infection cases are relatively rare, it remains a persistent foodborne pathogen detected across the food chain (Guo et al., 2023; Li et al., 2025) and capable of causing severe invasive disease (Sevellec et al., 2020; Luo et al., 2022). Given the resistance of certain strains to third-generation cephalosporins, carbapenems are commonly used for the treatment of severe infections in clinical settings (Lin et al., 2022), making the emergence of CRSA in this serovar a clinical concern. Despite this, no carbapenem-resistant *S*. Derby strain has been documented.

This study reports and characterizes the first identified carbapenem-resistant *S*. Derby isolates. We employed whole-genome sequencing (WGS) and phenotypic assays to elucidate its resistance mechanism, investigate its potential origins, and assess its dissemination risk. Meanwhile, this study conducts an evolutionary analysis comparing this strain with other carbapenemase-producing *Salmonella* in the database to understand the spread of CRSA. Our findings signal the expansion of carbapenem resistance into a new *Salmonella* lineage and provide essential data for updating risk assessments and clinical guidance.

## Materials and methods

### Bacterial isolates phenotypes confirmation

Carbapenem-resistant *S*. Derby isolate CS_CRSA and *Escherichia coli* isolate CS_CREco were isolated from rectal swab specimens from the hematology department of a tertiary hospital in Shandong Province, China. Specimens were inoculated onto blood agar, MacConkey, and Salmonella-Shigella (SS) plates and incubated at 37 °C under 5% carbon dioxide for 18–24 h. The isolates were identified by matrix-assisted laser desorption ionization time-of-flight mass spectrometry (MALDI-TOF; Bruker Daltonik, Bremen, Germany). The antimicrobial susceptibility test (AST) of general antibiotics was performed by using the MicroScan Walkway 96 system (Siemens Healthcare Diagnostics, CA, USA). *E. coli* ATCC25922 was used as the control strain, and the AST results were interpreted according to the Clinical and Laboratory Standards Institute guidelines (CLSI, M100-S34). The carbapenemase type was determined using the colloidal gold immunochromatography method (Dynamiker Biotechnology, Tianjin, China).

### Pulsed field gel electrophoresis

Pulsed-field gel electrophoresis (PFGE) was employed to ascertain the plasmid carriage among isolates and conjugation transfer. The S1 enzyme (Takara, Cina) was used for genome digestion, and the parameters for the CHEF MAPPER XA apparatus (Bio-Rad, USA) were set following the previously described elsewhere (Hao et al., 2020).

### Growth curves

The growth rate was monitored by changes in optical density at 620nm (OD620) over 20h. The single colony of isolates was inoculated in LB broth at 37°C overnight with shaking (200 rpm). Then the overnight cultures were diluted 1:200 into fresh LB to OD_620_ of 0.01, and 200μL suspensions were added to a 96-well plate. The plate was incubated at 37 °C with shaking for 20h in the Multiskan spectrum microplate reader (Thermo, USA), and the absorbance of each well at 620nm was measured every 30min.

### Plasmid stability testing

Plasmid stability was assessed as previously described with minor modifications (Gao et al., 2020). A single isolated colony was subjected to serial passage over 10 days (approximately 200 generations) at 37 °C with shaking at 200 rpm. Each day, overnight cultures were diluted 1000-fold into fresh antibiotic-free LB broth, and the remainder was preserved in glycerol at –20 °C for long-term storage. After 10 days, cultures were serially diluted and plated onto both antibiotic-free LB agar and LB agar containing meropenem (2 mg/L). The *bla*_NDM-1_ retention rate was calculated as the ratio of colony counts on meropenem-containing agar to those on antibiotic-free agar. The experiment included three parallel control groups, and colony PCR was performed to confirm the presence of the *bla*_NDM-1_ gene.

### Conjugation assay

Two rounds of conjugation tests were carried out to confirm the transferability of carbapenem resistance plasmids in CS_CRSA and CS_CREco. In the first round of experiments, fresh cultures of the recipient strain *E. coli* J53 Azi^R^ and the donor strain *S*. Derby CS_CRSA or *E. coli* CS_CREco were mixed in normal saline at a ratio of 1:2, and then the mixture was dropped onto 5% sheep blood agar. After incubation at 37°C for 24 hours, the bacterial film was scraped and serially diluted. Serial dilutions were spread on LB agar with 150 mg/L azide and 2 mg/L meropenem to screen for conjugated isolate *E. coli* J53T containing *bla*_NDM_-positive plasmid. The second round of conjugation experiments used the donor strain *E. coli* J53T (CS_CRSA), and the clinically relatively sensitive *Salmonella enterica* serovar Typhimurium (*S.* Typhimurium) isolate CSSA as the recipient strain. The SS agar plate supplemented with 150 mg/L azide and 2 mg/L meropenem was used to screen for conjugates. PCR was used to detect *bla*_NDM-1_ genes of conjugants. The conjugation efficiency was determined by dividing the number of transconjugant cells by the number of recipient cells (Wang et al., 2024).

### DNA sequencing and bioinformatics

Genomic DNA was extracted using an E.Z.N.A ^®^ Bacterial DNA kit (Omega Bio-tek). The harvested DNA was subject to next-generation sequencing (NGS) using the Illumina HiSeq system (Illumina, San Diego, CA, USA) and long-read sequencing using the Oxford Nanopore (MinION system). The hybrid assembly was performed by Unicycler v0.5.0 (Wick et al., 2017). The whole-genome sequences (WGS) were annotated automatically by Prokka tool in Proksee (Grant et al., 2023), followed by manual curation. OriT Finder was used to determine the conjugation module (Li et al., 2018). ISFinder (https://isfinder.biotoul.fr/) and MobileElementFinder (https://cge.food.dtu.dk/services/MobileElementFinder/) were used to identify Mobile Elements. Plasmid similarity was examined by comparing it against the PLSDB plasmid database (Schmartz et al., 2022). Comparison between homologous plasmids was performed using BLASTn and illustrated by Easyfig v2.2.2 (Sullivan et al., 2011).

Complete-level assembly *Salmonella* genome sequences and related information, including the isolate time, region, source, and host, were collected using the NCBI Genome database and the Pathogens database, with the collection time ending on June 25, 2025. Antibiotic resistance and plasmid replicon genes were identified using the ResFinder and PlasmidFinder in the CGE tools platform (https://genomicepidemiology.org/services/). The Multilocus sequence typing (MLST) and serovar typing of *Salmonella* strains were predicted using PubMLST (https://cge.food.dtu.dk/services/SeqSero/), SeqSero1 Integrated in (https://usegalaxy.cn/), and SISTR (https://lfz.corefacility.ca/sistr-app/), respectively. The core genomic sequences of CPSA were analyzed using CSI Phylogeny 1.4, single-nucleotide polymorphisms (SNPs) were identified and matrix was generated, and a phylogenetic tree was constructed using altered FastTree (Kaas et al., 2014). The visualization and annotation of the phylogenetic tree were carried out using the ChiPlot (Xie et al., 2023). The relevant nucleotide sequences have been uploaded to the NCBI database.

## Result

### Timeline of patient treatment and strain isolation

**Figure 1:**
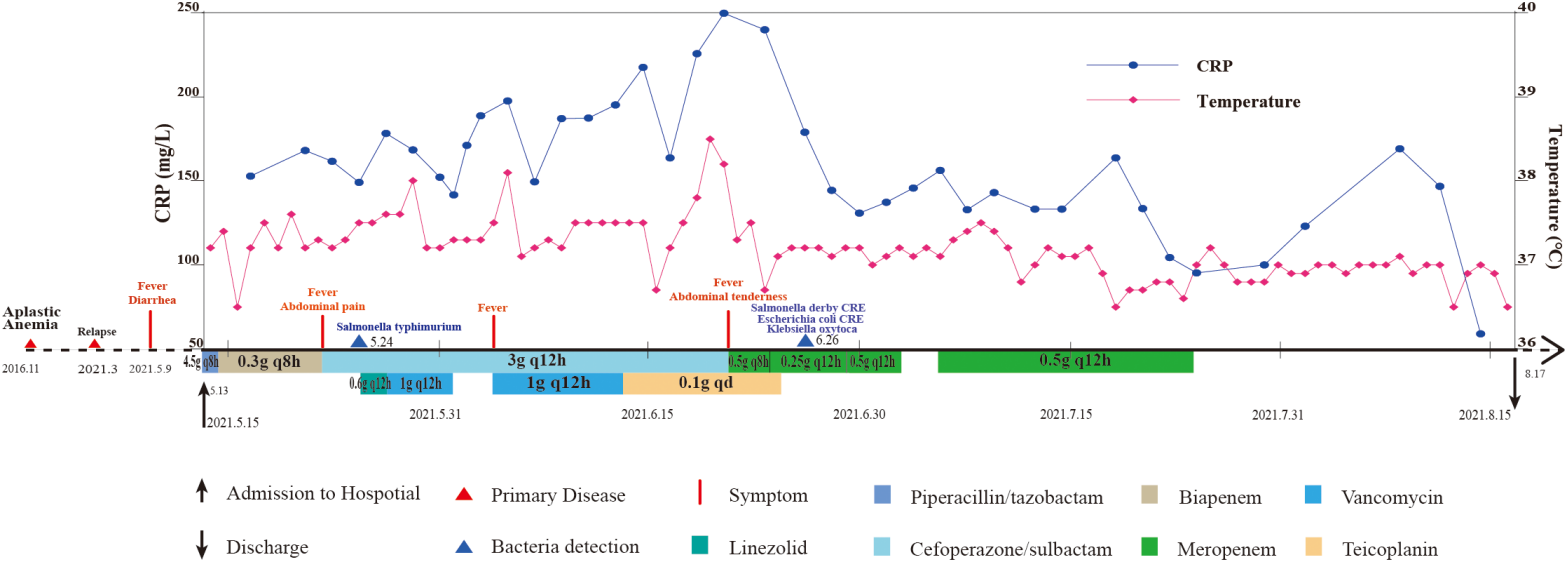
Timeline of diagnosis and treatment for patients with carbapenem-resistant *Salmonella enterica* serovar Derby.

In May 2021, a patient with aplastic anemia (AA) was admitted to the hematology-oncology department of a tertiary teaching hospital due to diarrhea and a fever after consuming cherries. Upon admission, the patient received cyclosporin and empirical antibiotic therapy, including the β-lactam combination piperacillin/tazobactam, the carbapenem agent biapenem, and other antibiotics. On hospital day 11, antibiotic-sensitive *S*. Typhimurium was isolated from the patient’s stool sample. Subsequently, on day 44, carbapenem-resistant *E. coli* and *S.* Derby were detected in the rectal swab (AST results are shown in **Supplementary Table 1**). From hospital day 71 until discharge, the patient’s antibiotic treatment was discontinued, with inflammatory markers and clinical condition stabilizing. More detailed information about the patient’s body temperature, C-reactive protein levels (CRP), and medication usage was illustrated in **Figure 1**. Additionally, during this patient’s hospitalization, no other bacterial isolates exhibiting a similar antibiotic resistance profile or carbapenemase phenotype were identified in clinical specimens submitted from the same ward.

### Comparative analysis of genomes and plasmids

**Figure 2:**
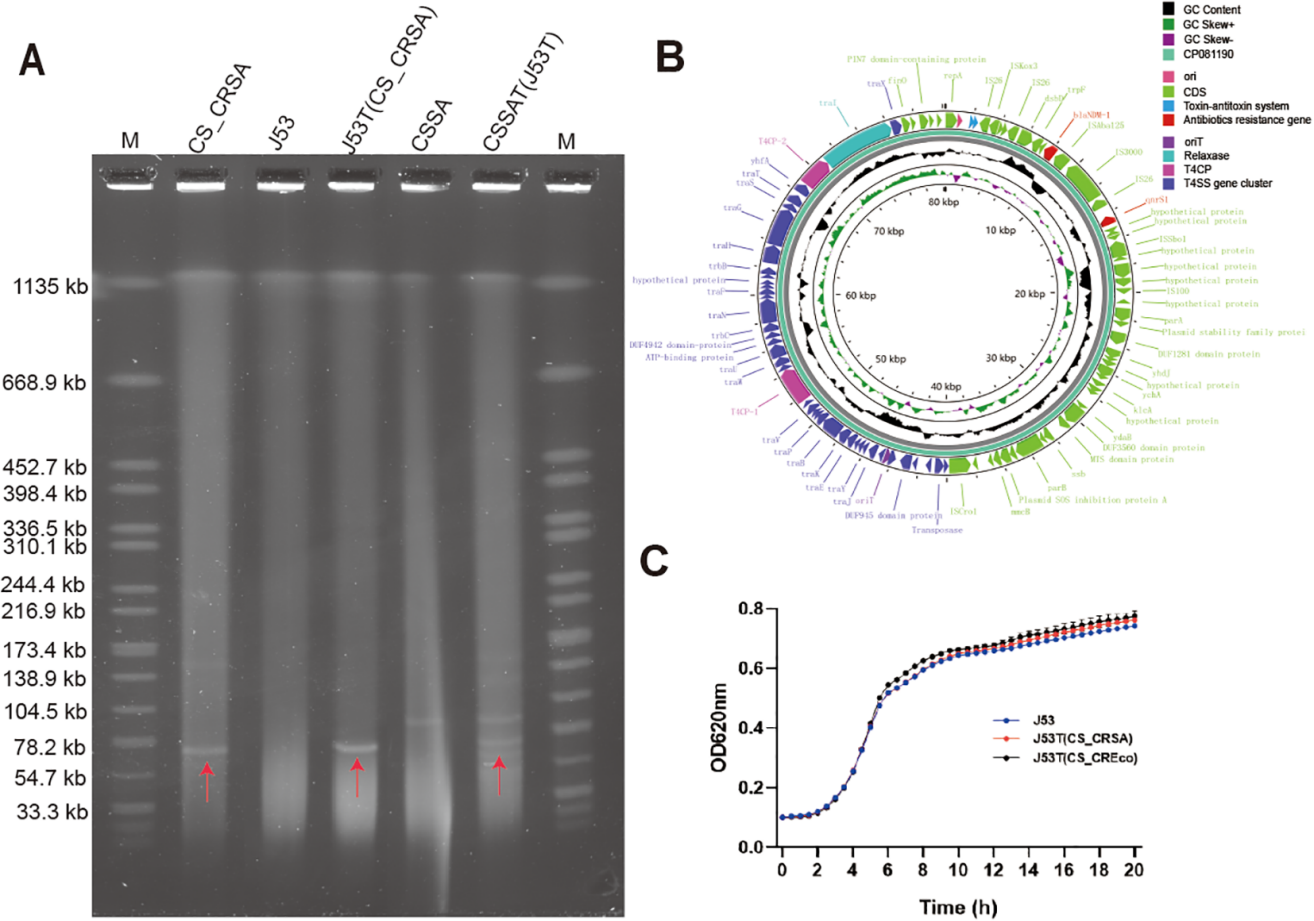
The genetic and transfer information of the plasmid pCS_CRSA in *Salmonella* Derby. **(A)** The S1 PFGE results of *S.* Derby and the conjugates obtained through two rounds of conjugation experiments. **(B)** Circular graph of the plasmid pCS_CRSA contained in *S.* Derby. **(C)** Growth rate curves of J53, J53T(CS_CRSA), and J53T(CS_CSEco).

**Figure 3:**
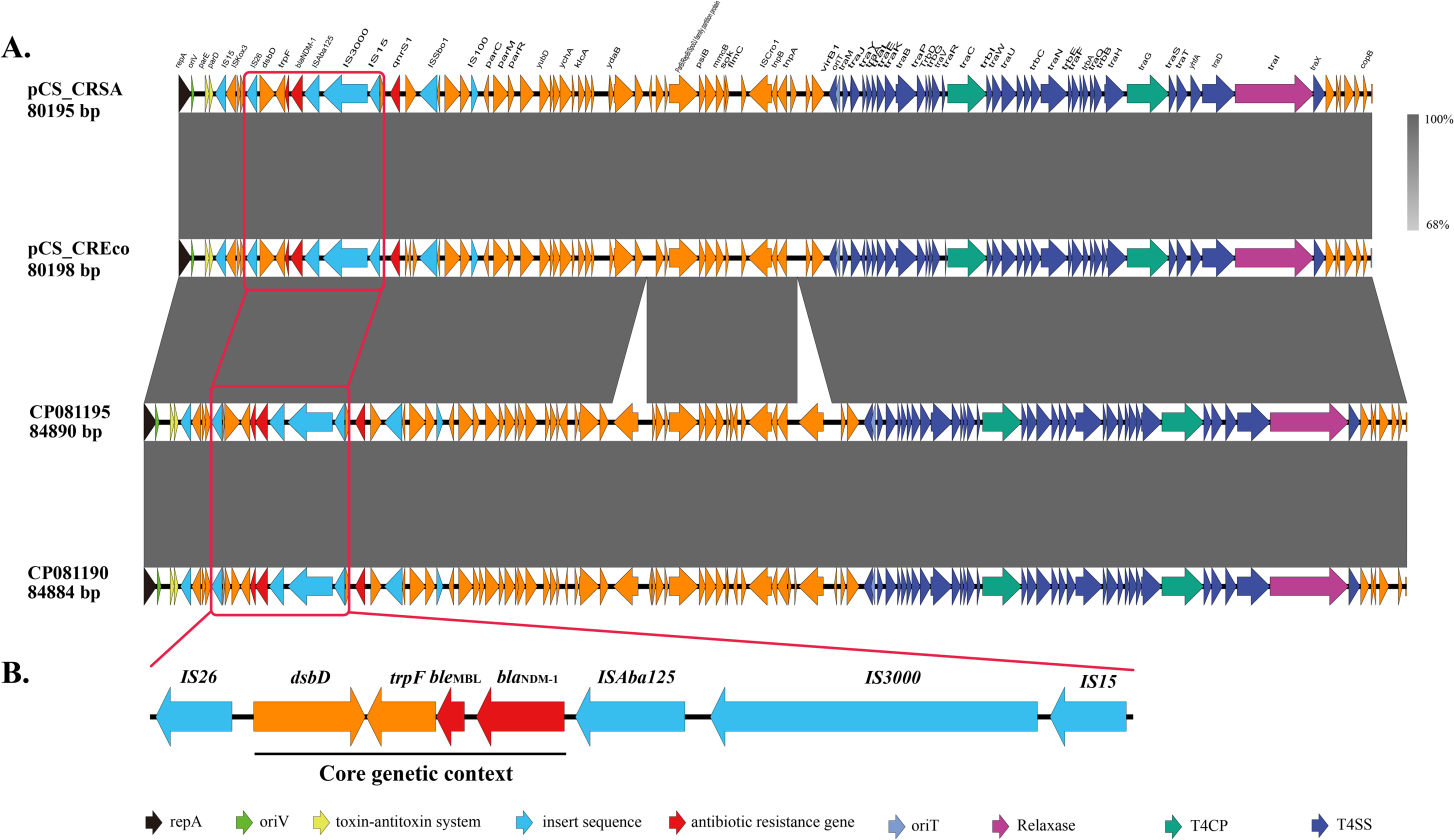
Linear characterization of plasmid pCS_CRSA. **(A)** Linear comparison of plasmid pCS_CRSA with its similar plasmids. **(B)** The genetic context of the *bla*_NDM-1_ gene.

After sequence assembly, complete genomes of both carbapenem-resistant *S*. Derby isolate CS_CRSA and *E. coli* isolate CS_CREco were obtained, with key replicon information summarized in **Table 1**. Both CS_CRSA and CS_CREco harbored nearly identical *bla*_NDM-1_-carrying plasmids, designated pCS_CRSA and pCS_CREco, which conferred carbapenem resistance. In addition to plasmid pCS_CREco, CS_CREco also contained other plasmids with or without additional resistance genes. Plasmids pCS_CRSA and pCS_CREco are IncFII-type plasmids with lengths of 80,195 bp and 80,198 bp, respectively, containing 91 coding sequences (CDS). They carry only two resistance genes, *bla*_NDM-1_ and *qnrS1*, and were predicted to harbor a complete conjugation transfer module (**Figures 2B**, **3**). In addition, the presence of multiple *Salmonella* pathogenicity islands (SPIs) was predicted in strain CS_CRSA.

**Table 1:** The genetic information of carbapenem-resistant Salmonella strains CS_CRSA and E. coli strains CS_CREco.

Replicons	Length (bp)	GC (%)	RepA	Conjugative function module	ARG
*Salmonella enterica* serovar Derby					
Chromosome	4884443	52.10%	—	—	aph(4)-Ia, aph(3’)-Ia, aac(3)-IV, aac(6’)-Ib-cr, aac(6’)-Iaa, blaOXA-1, fosA7, floR, catB3, OqxB, OqxA, qnrS2, ARR-3, sul2, sul3
pCS_CRSA	80195	52.40%	IncFII	OriT, relaxase, T4CP, T4SS	blaNDM-1, qnrS1
*Escherichia coli* CS-CREco					
Chromosome	4681426	50.80%	—	—	tet(A)
p2	246836	46.30%	IncHI2A	OriT, relaxase, T4CP, T4SS	aph(4)-Ia, aadA1, aadA2, aadA2b, aac(3)-IV, aadA8b, blaCTX-M-14, mcr-1.1, fosA3, cmlA1, OqxB, OqxA, sul1, sul2, sul3, dfrA12, bleO
p3	174486	49.70%	IncFIB/IncFIC	OriT, relaxase, T4CP, T4SS	—
pCS_CREco	80198	52.40%	IncFII	OriT, relaxase, T4CP, T4SS	blaNDM-1, qnrS1
p41k	41006	52.10%	IncX1	OriT	aadA16, aph(3’)-Ia, mph(A), floR, ARR-3, sul1, dfrA27

Using the online BLAST tool, pCS_CRSA was found to be highly similar to plasmids from *E. coli* strains 1.3GPP10-2 (OR965474.1), YZ22PE67 (CP171875.1), YZ22PE68 (CP171870.1), and EC1722-1 (CP081195.1), with coverage and identity values of 100%/99.98%, 100%/99.97%, 100%/99.96%, and 100%/99.97%, respectively. These *E. coli* strains were isolated from pork in a food market, pig feces, and pediatric stool samples, with geographical origins spanning Sichuan, Jiangsu, and Zhejiang, three provinces in China. Additionally, the plasmid (CP081190) harbored by *S.* Typhimurium sg1722–2 was almost identical to that in *E. coli* EC1722-1, both strains were isolated from pediatric stool samples in a hospital in Dongyang, Zhejiang Province. The discovery of pCS_CRSA-like plasmids in bacterial species beyond *S.* Derby raises the possibility that such plasmids may disseminate across regions and bacterial species. The genetic context of the *bla*_NDM-1_ locus was identified as IS26-*dsbD*-*trpF*-*ble*_MBL_-*bla*_NDM-1_-ISAba125-IS3000-IS15 (**Figure 3**), which forms a composite transposon, suggesting its potential for mobilization into various pathogens via transpositional recombination. BLAST analysis of this fragment also revealed its presence in various bacterial species, including *E. coli*, *Klebsiella pneumoniae*, and *Enterobacter hormaeche.*

### Transferability, growth cost, and stability of *bla*_NDM-1_ carrying plasmid

*In vitro* solid conjugation assays demonstrated that the plasmid carrying *bla*_NDM-1_ was transferred from strain CS_CRSA and strain CS_CREco to *E. coli* J53 at frequencies of (4.50 ± 1.29)×10^–2^ and (3.17 ± 0.74)×10^-1^, respectively (**Figure 2A**; **Supplementary Figure 1**), conferring resistance of the recipient strain to cephalosporins, carbapenems, and fluoroquinolones (**Supplementary Table 1**). *In vitro* growth assays showed that plasmid pCS_CRSA did not impose a fitness cost on the J53 strain (**Figure 2C**). Furthermore, the plasmid and the resistance gene it carries were stably maintained in the bacterial strains, with *bla*_NDM-1_ gene loss rates observed in only 3.57% for CS_CRSA and 2.00% for CS_CREco after 200 generations of continuous *in vitro* culture.

### The overview and phylogenetics of *Salmonella* producing carbapenemase

**Figure 4:**
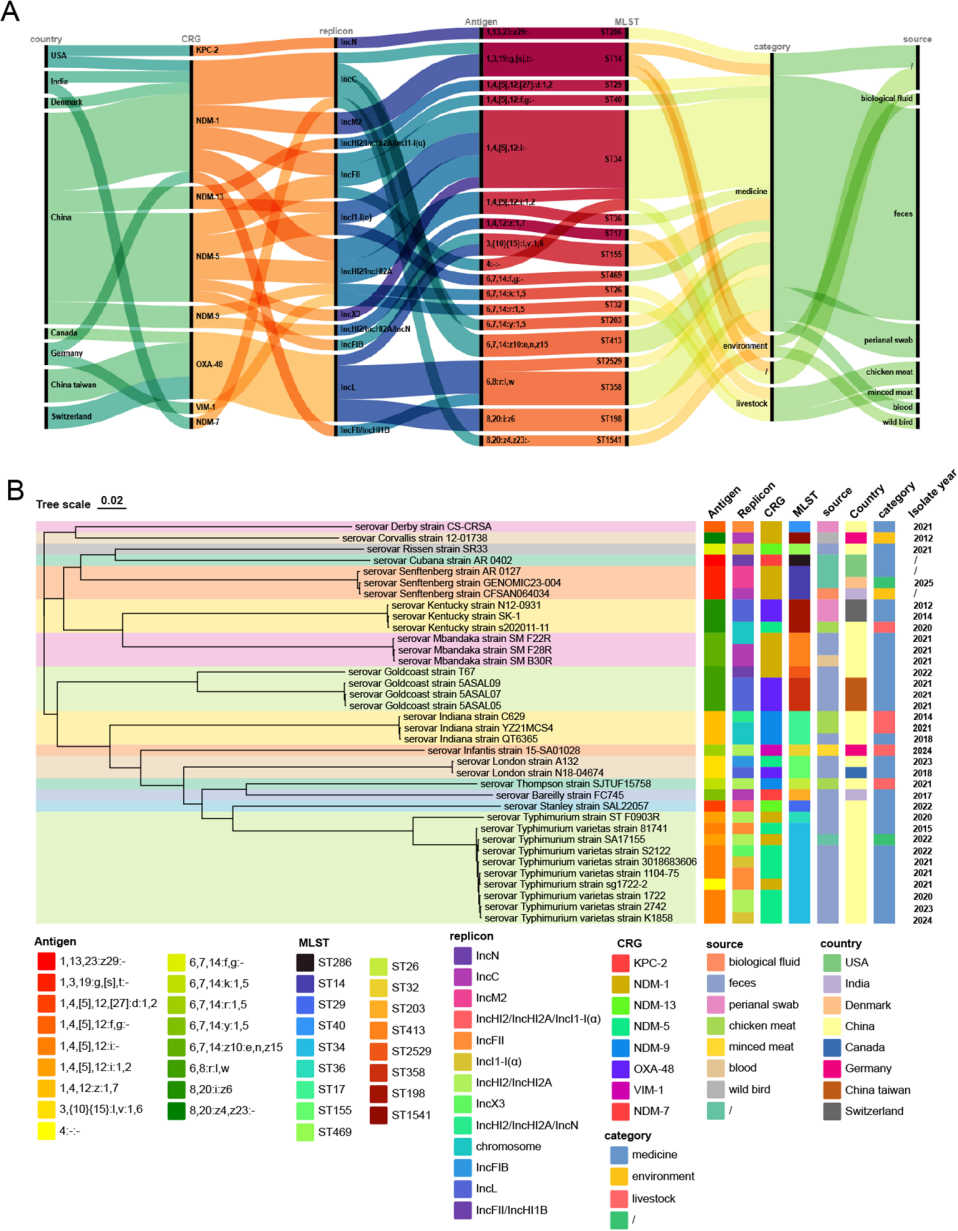
The genetic information and distribution of carbapenem-resistant *Salmonella.***(A)** Cumulative sum chart of characteristics related to carbapenem-resistant *Salmonella.***(B)** Phylogenetic analysis of carbapenem-resistant *Salmonella* species.

To comprehensively understand the global landscape of carbapenemase-producing *Salmonella* and contextualize the isolate *S.* Derby CS_CRSA, we analyzed fully assembled *Salmonella* genomes from the NCBI Genome and Pathogens databases up to June 25, 2025, and identified 36 carbapenemase-producing strains, including CS_CRSA from this study, with the earliest isolation dating back to 2012. Carbapenemases were predominantly of the NDM type (29/36), with NDM-1 being the most common subtype (13/29). The vast majority of carbapenemase genes were located on plasmids (88.89%, 32/36), primarily on IncH-related plasmids (31.3%, 10/32), followed by IncL (18.8%, 6/32) and IncC (15.6%, 5/32) types; a minority were located on the chromosome (11.1%, 4/36). These CPSA isolates were recovered from multiple countries or regions, predominantly from mainland China (63.9%, 23/36), all of which produced NDM-type carbapenemase. Most strains were clinically relevant (79.4%, 27/34), while a minority were associated with environmental (2/34) or livestock (5/34) sources. A total of 17 serovars were identified, mainly Typhimurium type and its variants (27.8%, 10/36), with the monophasic 1,4,[5],12:i:- (ST34) being the most common. Other serovars, such as Goldcoast, Mbandaka, and Senftenberg, were relatively rare (**Figure 4A**). Phylogenetic analysis revealed that the 36 CPSA strains emerged sporadically (**Figure 4B**). However, clonal dissemination was observed among NDM-1-producing serovar Mbandaka strains from mainland China and OXA-48-producing serovar Goldcoast strains from Taiwan, China (**Supplementary Figure 2**), and both clonal disseminations were clinically relevant. Notably, the *bla*_NDM-1_ gene of the Mbandaka serotype strain F22R was chromosomally located, and its flanking genetic context was identical to that of the plasmid-borne *bla*_NDM-1_ genes in strains F28R and F30R. Additionally, two Senftenberg serovar strains isolated from India and Denmark differed by only two SNPs, suggesting possible cross-border transmission (**Figure 4B**; **Supplementary Figure 2**).

## Discussion

This study provides the first systematic report and in-depth mechanistic analysis of a rare carbapenem-resistant *S.* Derby isolate. *S.* Derby is commonly associated with food-producing animals and generally considered to be of low virulence due to the absence of key virulence determinants, such as stress proteins, antivirulence factors, toxins, immune evasion genes, secretion systems, and fimbrial and non-fimbrial adhesins, often resulting in subclinical mild infection (Diemert and Yan, 2020; Tambassi et al., 2020). The isolate in this case belonged to ST40, one of the most prevalent lineages identified in human infections, and carried SPIs capable of modulating host immune responses and inducing inflammation, potentially leading to severe invasive infections in immunocompromised individuals (Vazquez et al., 2023). This pathogenicity was also manifested in the patient’s course of this case. Following an initial infection with an antibiotic-sensitive *S*. Typhimurium and treatment, this patient still developed marked systemic inflammation (elevated CRP and fever). Subsequently, a carbapenem-resistant *S*. Derby strain was isolated from a rectal swab. After the administration of meropenem, the patient’s clinical condition improved. Although the observed response appears paradoxical, the clinical improvement after full-dose meropenem therapy can be reasonably explained. This may be attributed to the renal impairment likely induced by cyclosporine use, which altered the pharmacokinetics of meropenem. Coupled with the relatively low meropenem minimal inhibitory concentration (4mg/L) of the isolate, these factors likely allowed the serum drug concentration to reach and maintain levels sufficient for bacterial suppression. The present case highlights that carbapenem resistance has emerged in traditionally considered rare and less virulent *S*. Derby. Greater clinical attention should therefore be paid to the resistance of such isolates, with enhanced surveillance and preventive measures in high-risk patient populations.

The carbapenem resistance in CS_CRSA was mediated by the *bla*_NDM-1_-carrying plasmid pCS_CRSA. Although environmental screening was not performed, the absence of any isolates with a similar resistance profile from other patients in the same ward throughout the prolonged hospitalization period suggests that a hospital-acquired transmission might have been less likely. Instead, this resistant isolate may have either been pre-existing in the patient’s gut microbiota or emerged during treatment through the horizontal transfer and selection of resistance plasmids among commensal bacteria. The pCS_CRSA plasmid was identified in both CS_CRSA and CS_CREco isolates, exhibiting features that facilitate dissemination, such as high stability in bacterial hosts and efficient conjugative transfer. Furthermore, homology analysis revealed that plasmids closely related to pCS_CRSA have been widely distributed in various bacterial hosts across multiple regions, supporting their potential to spread beyond a single host or environment. Based on these observations, we infer that the pCS_CRSA plasmid in CS_CRSA was likely acquired through horizontal transfer. Although no evidence currently indicates clonal spread of strains carrying this plasmid, its broad host adaptability and potential for horizontal transfer may contribute to an increased risk of carbapenem resistance dissemination among *Enterobacteriaceae* in clinical settings.

Phylogenetic analysis of CPSA strains reveals that these strains were mostly distributed sporadically, with carbapenemase genes mainly plasmid-borne. This pattern suggests that resistance acquisition mostly results from independent events rather than clonal expansion, likely driven by local antibiotic selection pressure or sporadic horizontal gene transfer. However, clonal dissemination has been observed in some regions for Mbandaka (Song et al., 2025) and Goldcoast (Chen et al., 2022) serotypes. Notably, some Mbandaka strains have undergone IS26-mediated chromosomal integration of the *bla*_NDM-1_ gene from plasmids (Song et al., 2025), indicating that these resistant clones have acquired enhanced adaptability and transmission capacity, enabling sustained inter-host colonization and spread. Of particular concern is the molecular evidence of international dissemination among some resistant clones, implicating cross-border human mobility, food trade, or travel in the global spread of resistance (Mthembu et al., 2021). Additionally, some CPSA isolates have been linked to food-producing animals and environmental sources. As a major foodborne pathogen, *Salmonella* can persistently colonize the intestines of food animals and contaminate animal-derived products. The prolonged misuse of antibiotics in animal husbandry has further promoted the emergence and intestinal colonization of multidrug-resistant *Salmonella* strains (Oh et al., 2025). These resistant *Salmonella* strains can circulate persistently among animal populations and enter the external environment through inadequately treated manure, contaminated water, or food products, thereby spreading to humans. Consequently, resistant *Salmonella* originating from food animals and the environment has become a significant source of community-acquired drug-resistant infections.

Compared to TS, NTS, such as the *S*. Derby strain in this study, generally exhibit lower virulence and more frequently cause self-limiting gastrointestinal infections or mild illnesses, including subclinical infections and asymptomatic carriage. Although these presentations are less severe at the individual level, subclinical NTS infections and asymptomatic carriage can have substantial public health implications: asymptomatic carriers may serve as reservoirs facilitating pathogen spread; low-virulence strains can cause severe disease in immunocompromised hosts; and persistently colonizing strains may transfer antimicrobial resistance genes to more virulent pathogens (Diemert and Yan, 2020). Carbapenems represent one of the last-line treatments for invasive NTS infections, for instance, sepsis and meningitis, or multidrug-resistant bacterial infections. Widespread dissemination of carbapenem resistance in NTS would severely compromise clinical management, potentially leading to increased treatment failures and mortality. Although growing numbers of reports on carbapenem-resistant NTS (Deng et al., 2024; Ke et al., 2024b; Zeng et al., 2024), these cases likely represent only the tip of the iceberg. As a major foodborne pathogen, recent evidence suggests that invasive NTS strains are increasingly transmitted through person-to-person contact, with gastrointestinal cases and asymptomatic carriers playing key roles in this dynamic (Zhou et al., 2025). Given that the gut microbiome serves as a reservoir for antimicrobial resistance (Anthony et al., 2021), interpersonal transmission mediated by intestinal carriers will inevitably facilitate more dynamic and unpredictable horizontal transfer of resistance genes, accelerating the emergence of resistant NTS. Furthermore, the detection of CRSA in animals and the environment underscores that clinical infection control alone is insufficient to curb resistance spread.

Therefore, a One Health strategy integrating genomic surveillance of human, animal, and environmental samples is essential. As demonstrated in this study, the resistance plasmid pCS_CRSA and its homologous variants have been detected in isolates from diverse sources, including human specimens, animal products, the market, and animal waste. This provides relatively clear targets for curbing the dissemination of such plasmids under the “One Health” surveillance framework. At the source level, surveillance should be expanded to systematically screen for carbapenem-resistant *Enterobacteriaceae* (CRE) in environments such as farms, slaughterhouses, food processing facilities, and wet markets. Based on surveillance findings, targeted enhanced disinfection should be implemented in transmission hotspots, along with further standardization and optimization of antimicrobial use in veterinary practice. At the human level, active screening for carriage of CRE should be conducted among high-risk occupational groups, such as livestock workers, slaughterhouse employees, market vendors, and hospitalized patients. For identified carriers, quarantine measures and health education should be provided, emphasizing personal hygiene and contact precautions to reduce secondary transmission of CRE within household and community settings.

## Conclusion

In conclusion, this study reports a rare case of carbapenem-resistant *S.* Derby, mediated by a conjugative *bla*_NDM-1_-carrying plasmid with high transfer potential. Although horizontal plasmid transfer is the most plausible origin of this resistant strain, direct supporting evidence—such as temporal genomic or longitudinal microbiome data—is lacking. In addition, another limitation of this study is that our assessment of plasmid dissemination relies primarily on sequence homology rather than established transmission links. Nevertheless, this case indicates that carbapenem resistance is extending to less common serovars of *Salmonella*. CRSA clonal dissemination in certain serovars is concerning, particularly in light of increasing person-to-person transmission of resistant NTS. Furthermore, as an important foodborne pathogen, it is essential to adopt a One Health framework to monitor the prevalence and transmission dynamics of CRSA and to implement evidence−based control measures.

## Data Availability

The complete genome sequences of CS_CRSA and CS_CREco described in this study have been deposited in the NCBI under the BioProject accession number PRJNA1365005. The authors confirm that the other data supporting the results of this study are available within the article and its **Supplementary Materials**. All of the associated data are available from the corresponding author upon reasonable request.

## Supplementary material

Supplementary Figure 1 The S1 PFGE results of the strains CS_CRSA and CS_CREco and their corresponding conjugates.

Supplementary Figure 2 SNP number Matrix Diagram of Carbapenem-resistant *Salmonella*.
