# Supplementary figures and images for: Carbapenem-resistant Salmonella Derby harboring a plasmid carrying blaNDM-1 from a clinical case in China
Source: Front Cell Infect Microbiol. 2026 Mar 9;16:1765519. doi: 10.3389/fcimb.2026.1765519 (PMC13006696; doi:10.3389/fcimb.2026.1765519)

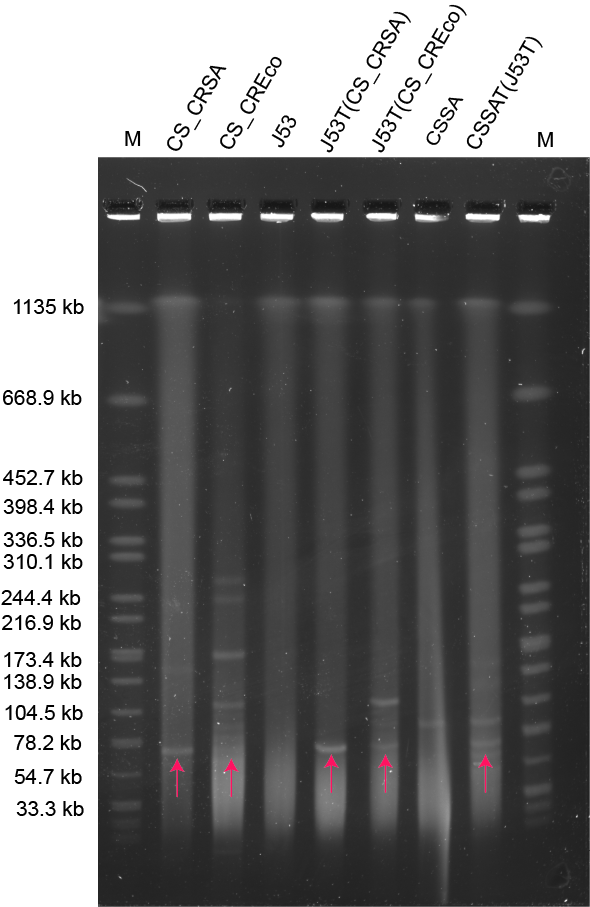

Supplement: Supplementary Figure 1 — The S1 PFGE results of the strains CS_CRSA and CS_CREco and their corresponding conjugates. [file Image1.tif]

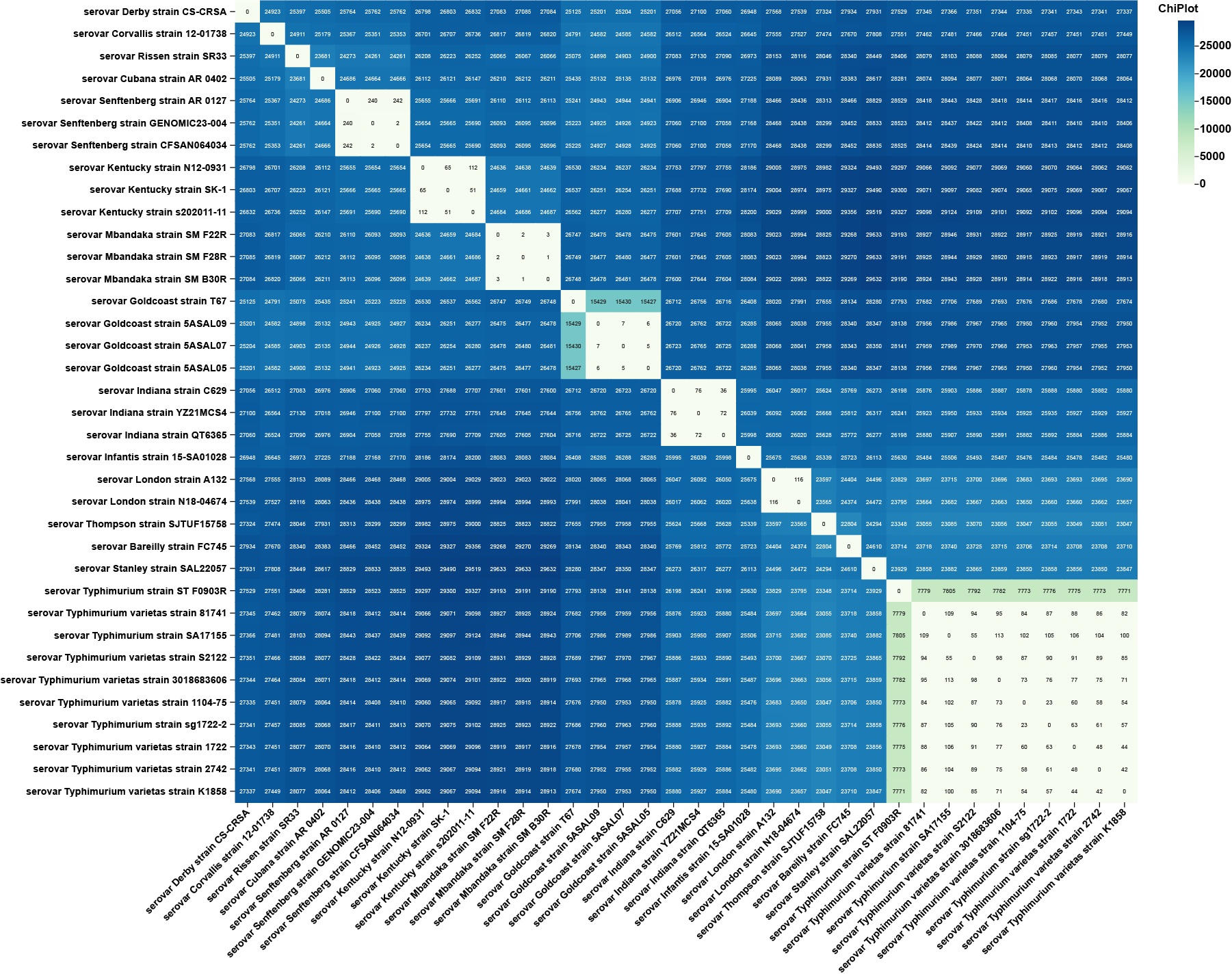

Supplement: Supplementary Figure 2 — SNP number Matrix Diagram of Carbapenem-resistant Salmonella. [file Image2.tif]
