# Supplementary material for: Carbapenem-resistant Salmonella Derby harboring a plasmid carrying blaNDM-1 from a clinical case in China
Source: Front Cell Infect Microbiol. 2026 Mar 9;16:1765519. doi: 10.3389/fcimb.2026.1765519 (PMC13006696; doi:10.3389/fcimb.2026.1765519)
Supplement: Supplementary file 3 [file Table1.docx]

Supplement Table 1: The antimicrobial susceptibility of the strains involved in the conjugation transfer test.

| **Antimicrobial** | **CS_CRSA** | **CS_CREco** | **J53** | **CS_CRSA-T(J53)** | **CS_CREco-T(J53)** | **CSSA** | **CSSAT** |
| --- | --- | --- | --- | --- | --- | --- | --- |
| **Ampicillin** | **>16** | **>16** | **≤8** | **>16** | **>16** | **>16** | **>16** |
| **Piperacillin** | **>64** | **>64** | **≤16** | **>64** | **>64** | **>64** | **>64** |
| **Ampicillin/Sulbactam** | **>16/8** | **>16/8** | **4/2** | **>16/8** | **>16/8** | **16/8** | **>16/8** |
| **Amoxicillin/Clavulanic** | **>16/8** | **>16/8** | **8/4** | **>16/8** | **>16/8** | **8/4** | **>16/8** |
| **Piperacillin/Tazobactam** | **＞64/4** | **＞64/4** | **≤4/4** | **＞64/4** | **＞64/4** | **≤4/4** | **＞64/4** |
| **Cefoxitin** | **＞16** | **＞16** | **≤8** | **＞16** | **＞16** | **≤8** | **＞16** |
| **Aztreonam** | **≤4** | **＞16** | **≤4** | **≤4** | **≤4** | **≤4** | **≤4** |
| **Cefazolin** | **＞16** | **>16** | **≤2** | **>16** | **>16** | **4** | **>16** |
| **Cefuroxime** | **＞16** | **>16** | **≤4** | **>16** | **>16** | **≤4** | **>16** |
| **Ceftriaxone** | **＞32** | **＞32** | **≤1** | **＞32** | **＞32** | **≤1** | **＞32** |
| **Cefotaxime** | **＞32** | **＞32** | **≤1** | **>32** | **＞32** | **≤1** | **＞32** |
| **Ceftazidime** | **＞16** | **＞16** | **≤2** | **＞16** | **＞16** | **≤1** | **＞16** |
| **Cefepime** | **＞16** | **＞16** | **≤2** | **＞16** | **＞16** | **≤2** | **＞16** |
| **Imipenem** | **8** | **＞8** | **≤0.5** | **＞8** | **8** | **≤0.5** | **8** |
| **Meropenem** | **4** | **＞8** | **≤0.5** | **＞8** | **＞8** | **≤0.5** | **4** |
| **Amikacin** | **≤16** | **≤16** | **≤16** | **≤16** | **≤16** | **≤16** | **≤16** |
| **Levofloxacin** | **4** | **＞4** | **≤0.25** | **1** | **1** | **0.5** | **1** |
| **Sulfamethoxazole/trimethoprim** | **≤2/38** | **＞2/38** | **≤2/38** | **≤2/38** | **≤2/38** | **≤2/38** | **≤2/38** |
| **Tetracycline** | **≤4** | **＞8** | **≤4** | **≤4** | **≤4** | **＞8** | **＞8** |
| **Tigecycline** | **≤2** | **≤2** | **≤2** | **≤2** | **≤2** | **≤2** | **≤2** |

Supplement Table 2: Genetic and isolation information of carbapenem-resistant Salmonella of different serotypes.

| **Serovar** | **Replicon** | **Antigen** | **CRG** | **MLST** | **Isolation source** | **Country** |
| --- | --- | --- | --- | --- | --- | --- |
| Typhimurium(2) | IncHI2/IncHI2A | 1,4,[5],12:i:1,2 | *bla*_NDM-1_ | ST36, ST34 | feces | China |
| Typhimurium varietas(8) | IncHI2/IncHI2A; IncFII, IncI1-I(α), IncX3 | 1,4,[5],12:i:-;  4:-:- | *bla*_NDM-5_, *bla*_NDM-1_ | ST34 | feces | China |
| Goldcoast(4) | IncFII/IncHI1B, IncL | 6,8:r:l,w | *bla*_NDM-1_, *bla*_OXA-48_ | ST2529, ST358 | feces | China; China Taiwan |
| Indiana Serovar(3) | chromosome, IncHI2/IncHI2A/IncN | 1,4,12:z:1,7 | *bla*_NDM-9_ | ST17 | chicken meat, feces | China |
| Kentucky(3) | chromosome, IncL | 8,20:i:z6 | *bla*_NDM-5_, *bla*_OXA-48_ | ST198 | chicken meat, perianal swab | China, Switzerland |
| London(2) | IncFIB, IncL | 3,10,15:l,v:1,6 | *bla*_NDM-5_, *bla*_OXA-48_ | ST155 | stool | China, Canada |
| Mbandaka(3) | chromosome, IncC | 6,7,14:z10:e,n,z15 | *bla*_NDM-1_ | ST413 | feces, blood | China |
| Senftenberg(3) | IncC, IncM2 | 1,3,19:g,[s],t:- | *bla*_NDM-1_ | ST14 | biological fluid, / | India, USA, Denmark |
| Bareilly(1) | IncC | 6,7,14:y:1,5 | *bla*_NDM-7_ | ST203 | feces | India |
| Corvallis(1) | IncC | 8,20:z4,z23:- | *bla*_NDM-1_ | ST1541 | wild bird | Germany |
| Cubana(1) | IncN | 1,13,23:z29:- | *bla*_KPC-2_ | ST286 | — | USA |
| Derby(1) | IncFII | 1,4,[5],12:f,g:- | *bla*_NDM-1_ | ST40 | perianal swab | China |
